# Unveiling the dynamic and thermodynamic interactions of hydrocortisone with β-cyclodextrin and its methylated derivatives through insights from molecular dynamics simulations

**DOI:** 10.1038/s41598-024-63034-7

**Published:** 2024-05-31

**Authors:** Roya Gholami, Khaled Azizi, Mokhtar Ganjali Koli

**Affiliations:** 1 Department of Chemistry, University of Kurdistan, Sanandaj, Iran; 2 Computational Chemistry Laboratory, Kask Afrand Exire Ltd., Sanandaj, Iran

**Keywords:** Inclusion complex, Cyclodextrin derivatives, Molecular dynamics simulation, Drug delivery systems, Thermodynamic properties, Conformational changes, Computational biophysics, Computational biology and bioinformatics, Molecular dynamics

## Abstract

Cyclodextrins (CDs) can enhance the stability and bioavailability of pharmaceutical compounds by encapsulating them within their cavities. This study utilized molecular dynamics simulations to investigate the interaction mechanisms between hydrocortisone (HC) and various methylated CD derivatives. The results reveal that the loading of HC into CD cavities follows different mechanisms depending on the degree and position of methylation. Loading into βCD and 6-MeβCD was more complete, with the hydroxyl groups of HC facing the primary hydroxyl rim (PHR) and the ketone side facing the secondary hydroxyl rim (SHR). In contrast, 2,3-D-MeβCD and 2,6-D-MeβCD showed a different loading mechanism, with the ketone side facing the PHR and the hydroxyl groups facing the SHR. The root mean square fluctuation (RMSF) analysis demonstrated that methylation increases the flexibility of CD heavy atoms, with 3-MeβCD and 2,3-D-MeβCD exhibiting the highest flexibility. However, upon inclusion of HC, 3-MeβCD, 2,3-D-MeβCD, 2-MeβCD, and 6-MeβCD showed a significant reduction in flexibility, suggesting a more rigid structure that effectively retains HC within their cavities. The radial distribution function revealed a significant reduction in the number of water molecules within the innermost layer of the methylated CD cavities, particularly in TMeβCD, indicating a decrease in polarity. The presence of HC led to the release of high-energy water molecules, creating more favorable conditions for HC loading. Conformational analysis showed that methylation caused a partial decrease in the area of the PHR, a significant decrease in the area of the middle rim, and a notable decrease in the area of the SHR. The loading of HC increased the area of the PHR in most derivatives, with the most pronounced increase observed in 2,6-D-MeβCD and 6-MeβCD. The analysis of interaction energies and binding free energies demonstrated that the binding of HC to methylated CD derivatives is thermodynamically more favorable than to βCD, with the strongest association observed for 6-MeβCD, 2-MeβCD, and 2,3-D-MeβCD.

## Introduction

Corticosteroids, a category of steroid hormones secreted by the adrenal cortex, include glucocorticoids and mineralocorticoids^1^. Glucocorticoids, of which hydrocortisone (HC) is a prime example, are important in the treatment of a variety of inflammatory and autoimmune diseases, including asthma, allergies, septic shock, rheumatoid arthritis, inflammatory bowel disease, and multiple sclerosis^2^. However, their clinical efficacy is hampered by adverse reactions associated with high doses (as seen in systemic vasculitis and SLE) and prolonged administration. These adverse effects include osteoporosis, skin atrophy, diabetes, abdominal obesity, glaucoma, cataracts, avascular necrosis, increased susceptibility to infection, growth retardation, and hypertension^2,3^. In addition, their therapeutic potential may be undermined by poor aqueous solubility, which affects their bioavailability and therapeutic efficacy^4^. To overcome these limitations, researchers have turned to Cyclodextrins (CDs) to form inclusion complexes with corticosteroids^5,6^.

CDs are cyclic oligosaccharides composed of glucose units arranged in a toroidal structure, creating a hydrophobic cavity capable of hosting hydrophobic drug molecules. Through host–guest interactions, CDs can encapsulate HC molecules, thereby improving their solubility, stability, and pharmacokinetic properties^7,8^.

Schönbeck et al.^9^ comprehensively analyzed the phase-solubility (PS) relationship of the HC/γ-cyclodextrin (γCD) system employing isothermal titration calorimetry (ITC), high-performance liquid chromatography (HPLC) for quantification, and precipitate composition analysis. ITC determined a 1:1 binding stoichiometry between HC and γCD with a precise binding constant (K11). The PS diagram exhibited a BS-type shape with three discernible regions. The initial solubility increase of HC was attributed to 1:1 complex formation, followed by a plateau due to the precipitation of a complex with approximately 1.5 γCDs per HC. Higher γCD concentrations led to a marked decrease in HC solubility due to the low solubility product (KS32) of the precipitating 3:2 γCD:HC complex.

Schwarz et al.^10^ investigated the solubilization of seven steroidal drugs (testosterone, estradiol, etc.) by six βCD derivatives. The anionic thioether heptakis-6-sulfoethylsulfanyl-6-deoxy-β-cyclodextrin (HSES) gave high steroid solubilities up to 19 mM, while the neutral thioethers heptakis-6-methylsulfanyl-6-deoxy-2-(2-(2-(2-methoxyethoxy)ethoxy)ethoxy)ethyl)]-βCD (HTMT) and heptakis-6-thioglyceryl-6-deoxy-βCD (HTG) exhibited selectivity for testosterone and estradiol, respectively. Solubilization occurred due to the inclusion of the steroid rings in the CD cavity. Further research could build upon these promising CD-steroid systems to enable improved delivery of hydrophobic steroidal drugs.

Kristmundsdóttir et al.^11^ developed a hydrocortisone mouthwash utilizing CDs to improve solubility and stability. The formulation demonstrated stability under storage and heating, effective antimicrobial preservation, and promising results in improving symptoms in 78% of 50 patients with oral inflammatory conditions. Further development of CD-enabled steroid formulations could provide convenient and effective treatments for oral diseases.

Hydrocortisone is frequently prescribed off-label for children, but appropriate pediatric formulations are lacking. As a result, hydrocortisone tablets are often manipulated by crushing or splitting to achieve lower doses, but this can lead to dosing errors or lack of bioequivalence compared to tablets^12^. To address this issue, Orlu-Gul et al.^13^ developed a reconstitutable oral solution with a 1:6 HC:hydroxypropyl-β-cyclodextrin (HPβCD) ratio for fast, complete solubilization. Key features included taste-masking with neotame and HPβCD for bitterness and stability, allowing flexible dosing with improved palatability and stability compared to compounded suspensions.

From an experimental viewpoint, guest-CD interactions are investigated using Isothermal Titration Calorimetry (ITC) for thermodynamic parameters, Thermogravimetric and Differential Thermal Analysis (TG/DTA) for thermal stability, Fourier Transform Infrared Spectroscopy (FTIR) for functional group identification, Proton Nuclear Magnetic Resonance (1H-NMR) for structural changes upon complexation^14^, and X-ray Crystallography for spatial arrangement and chemical bonding, all crucial for CD optimization in applications like drug delivery systems^15^. In this regard, computational methods such as molecular dynamics simulations offer significant advantages in studying drug-CD interactions and overall in drug delivery as well as other biological systems. They provide detailed, atomic-level insights into binding mechanisms, thermodynamic properties, and structural changes, enabling researchers to optimize drug formulations and predict biological behavior with increased accuracy and efficiency, ultimately advancing the field of personalized medicine^16–18^.

## Main strategy

**Figure 1:**
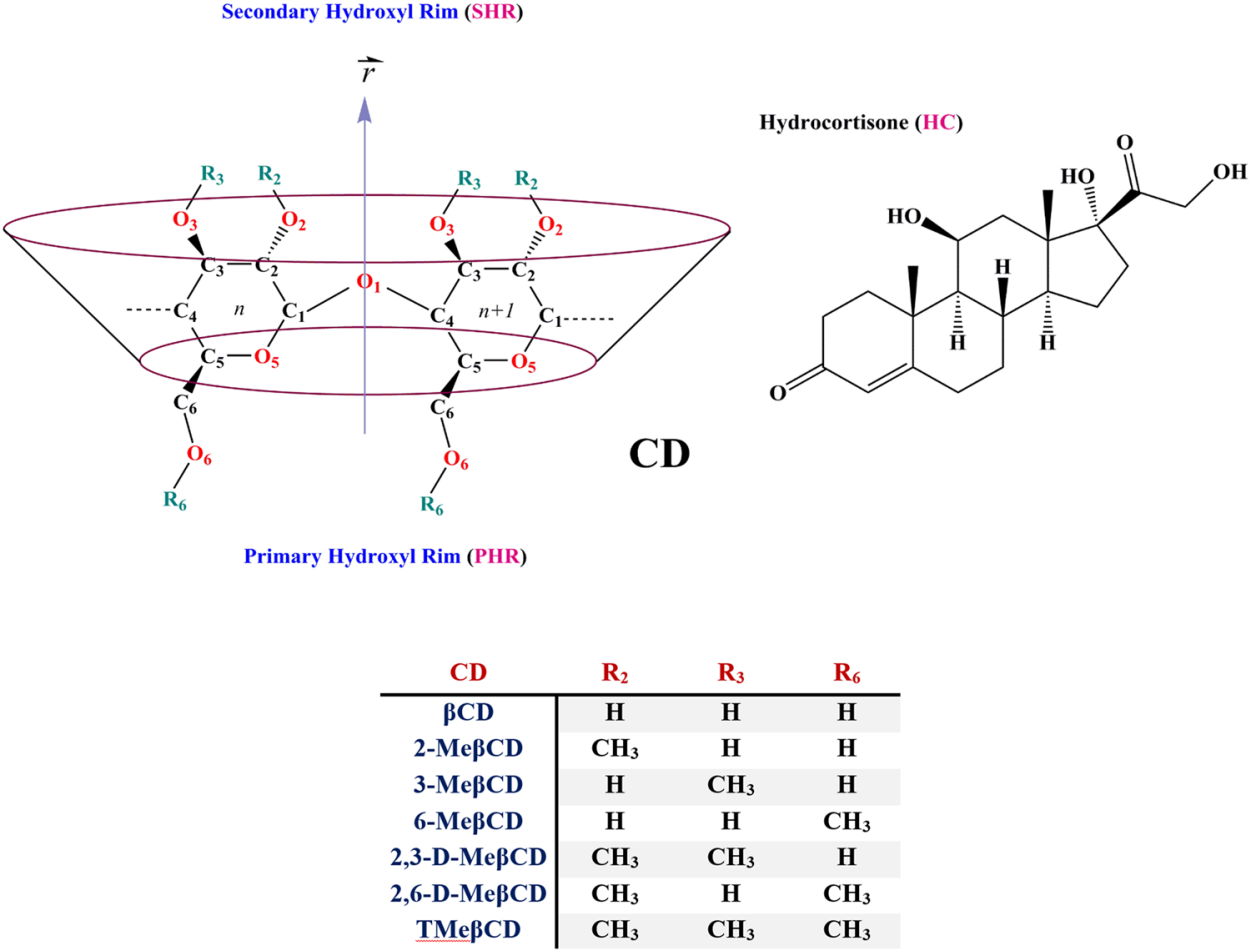
Molecular structure of Methylated β-cyclodextrin derivatives and Hydrocortisone.

Methylated β-cyclodextrin (MeβCD) offers enhanced drug delivery capabilities compared to β-cyclodextrin (βCD), including improved solubility, bioavailability, reduced toxicity, targeted delivery, controlled release, and enhanced barrier penetration, rendering it advantageous for inclusion in drug delivery systems^19–21^. On the other side, the interaction between hydrocortisone and CDs, particularly methylated-CD, is significant due to its impact on the solubility, stability, and bioavailability of hydrocortisone. CDs form inclusion complexes with steroids like hydrocortisone, enhancing their water solubility and solution stability. For instance, hydroxypropyl-βCD (HPβCD) increases hydrocortisone bioavailability in aqueous humor and cornea^5^. MeβCDs form more soluble complexes with hydrocortisone compared to the native βCD^22^. MeβCDs decelerate the alkaline and acidic degradation of hydrocortisone, providing increased chemical stability^23^. The hydroxide ion-catalyzed rearrangement of hydrocortisone 17-butyrate into the 21-butyrate of lower biological activity was catalyzed by βCD and inhibited by dimethyl-βCD at pH 2–8^24^. This study aimed to investigate the well-characterized structures of MeβCD from both thermodynamic and structural perspectives and to assess their interactions with HC (Log *p* = 1.61, water solubility 0.311 mg/mL)^25–27^ for potential use in drug delivery systems. Different polymerization states of MeβCD, which are well-known in experimental research, were considered based on their degree of polymerization^28^, as illustrated in Fig. 1.

**Figure 2:**
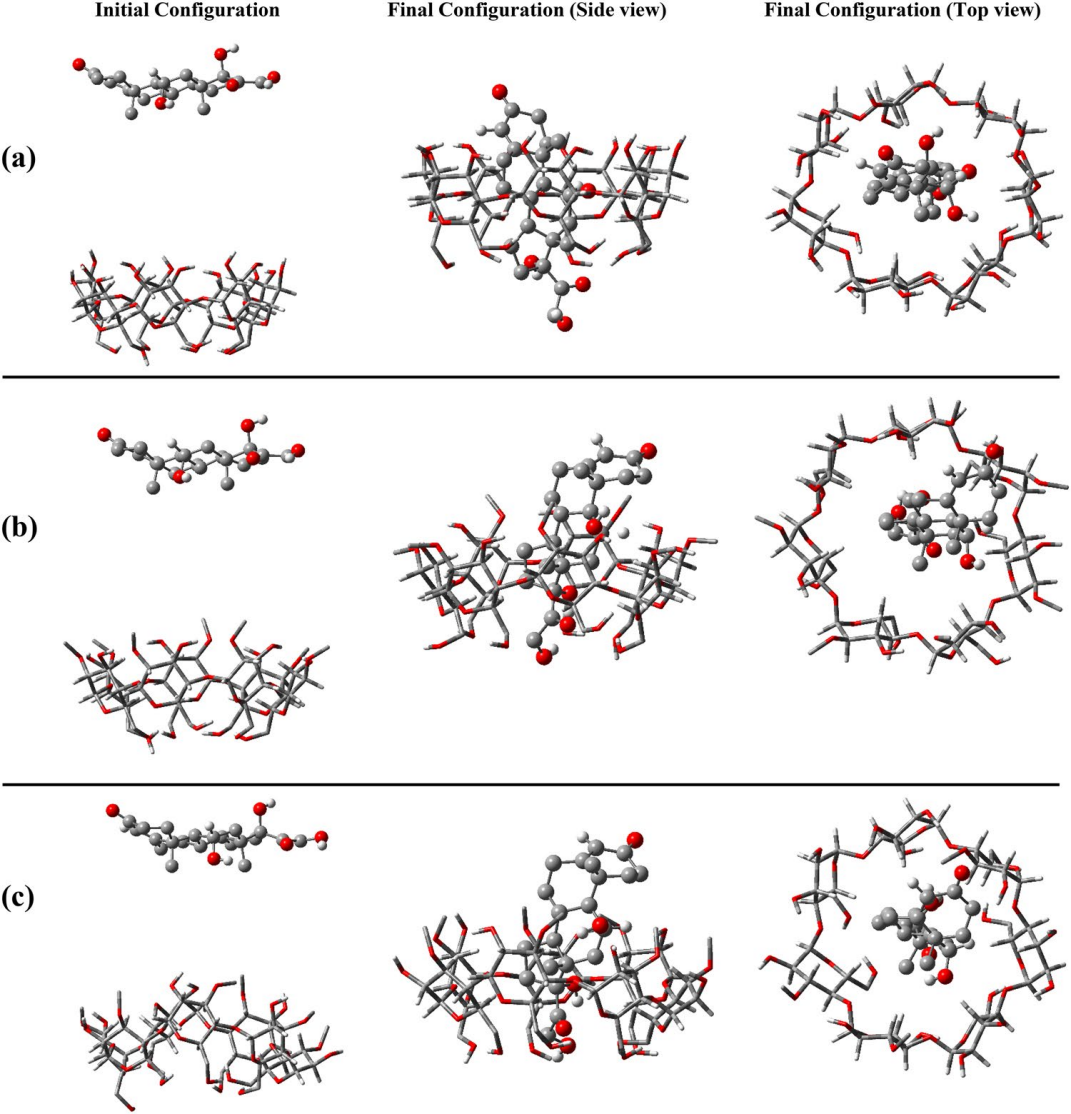

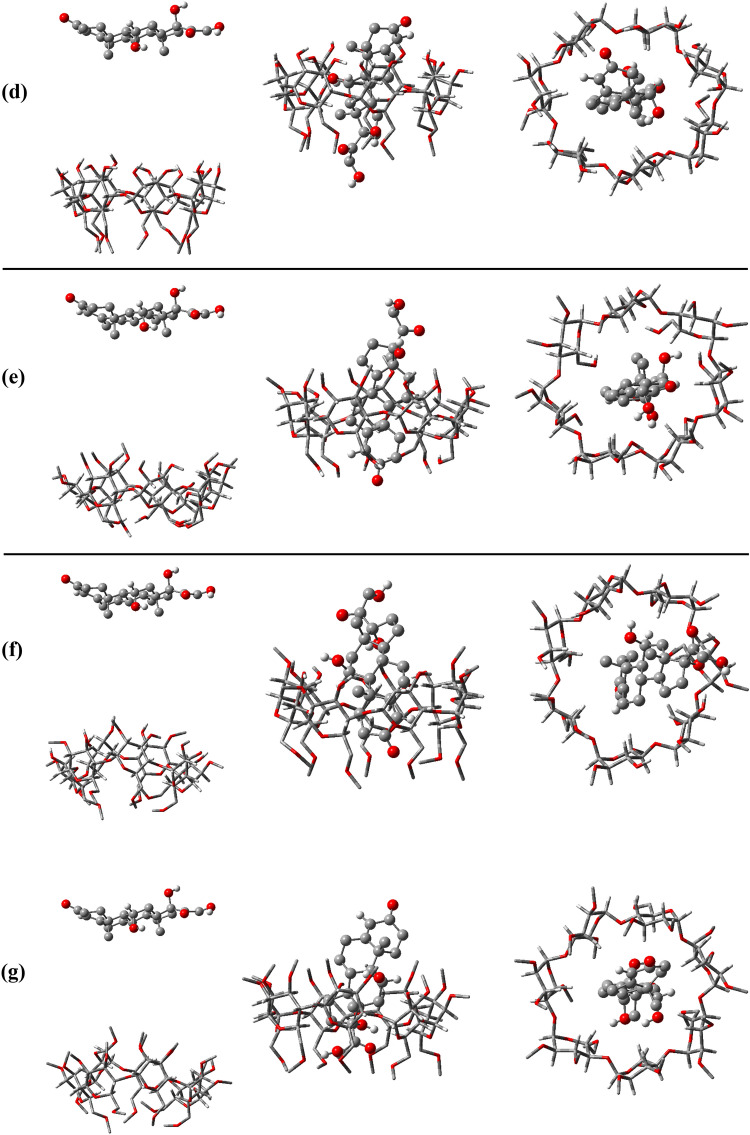
Snapshots of the initial and final configurations of hydrocortisone toward βCD (**a**), 2-MeβCD (**b**), 3-MeβCD (**c**), 6-MeβCD (**d**), 2,3-D-MeβCD (**e**), 2,6-D-MeβCD (**f**), and TMeβCD (**g**).

First, we simulated each MeβCD structure and βCD separately in water. Then, by placing an HC molecule 1.5 nm away from the secondary hydroxyl rim (SHR), we conducted simulations again to identify the potential of each of the polymerized states and to examine the interactions in the 1:1 stoichiometry without assuming any initial a priori inclusion, as shown in Fig. 2. This approach will illuminate the suitability of each structure for use in drug delivery systems, contributing to the development of more effective and targeted drug formulations.

## Computational details

### Molecular dynamics simulation

The molecular dynamics (MD) simulation was conducted using GROMACS 2022.2 software^29–31^, employing the GROMOS 54a7 force field^32,33^ to model a binary mixture of CDs and HC immersed in a solvent of 4500 water molecules. A validation related to the force field has been conducted by comparing some of the fundamental experimental parameters associated with βCD, as depicted in Figure S1 and Table S1. The water molecules were represented using the extended simple point charge model (SPC/E)^34,35^. The simulation employed periodic boundary conditions in all three dimensions (xyz) to mimic bulk behavior. To eliminate unfavorable atomic interactions, a steepest descent energy minimization was employed^36^, and the systems underwent equilibration processes within the NVT and NPT ensembles for 1 ns and 9 ns, respectively. After achieving equilibration, a total simulation time of 200 ns was considered in the NPT ensemble for each system. Integration of Newton's equations utilized the leap-frog algorithm^37^ with a time step of 2 fs. Coordinates, velocities, energies, and log data were saved every 2.0 ps, with compressed coordinates stored for efficient analysis. Bonded interactions were maintained using the LINCS algorithm^38^ with all bonds constrained, while van der Waals (*vdW*) interactions were truncated at a cutoff radius of 1.2 nm. Coulombic (*Coul*) interactions were calculated via the Particle Mesh Ewald (PME) method^39^ with a real-space cutoff of 1.2 nm, complemented by a long-range dispersion correction. Temperature was controlled using the V-rescale technique^40^ with a time constant of 0.1 ps and a reference temperature of 300 K for each coupling group. Pressure coupling utilized the Parrinello-Rahman method^41^ with an isotropic pressure coupling scheme, a time constant of 2.0 ps, and a reference pressure of 1.0 bar, considering the compressibility of water set to 4.6 × 10^–5^ bar^-1^.

### Free energy computations

The solvation free energy of a solvated system was determined by evaluating the free energy difference in a transforming system as it moves between solvated and unsolvated states. This computational analysis involved the utilization of a coupling parameter (λ) in conjunction with the thermodynamic integration (TI) formula^42,43^.

1 $${\Delta G}_{AB}={\int }_{{\uplambda }_{A}}^{{\uplambda }_{B}}{\langle \frac{\partial H (\uplambda )}{\partial\uplambda }\rangle }_{{\uplambda }^{\prime}}d\uplambda$$

The Hamiltonian (H) was quantified as a function of the coupling parameter λ. This parameter serves as an indicator of the extent of transformation occurring between specific states, such as the transition from solvated to unsolvated states or between states A and B. The TI method relies on the λ-dependence of the Hamiltonian for solute–solvent interactions, which gradually varies between full interactions (corresponding to λ = 0) and no interaction (corresponding to λ = 1). In other words, the solute molecule is gradually made to disappear from the solution using the coupling parameter λ. The Bennett acceptance ratio method (BAR)^44^ was employed to assess variations in free energy across different λ values in Hamiltonians. A thermodynamic cycle utilizing alchemical free energy computations^45,46^ was utilized to ascertain binding free energy. The computation involved gradually reducing the potential energy of interaction between HC and its surroundings using a λ parameter spanning 0 to 1^47,48^. For calculating ΔG_solv_, 25 λ points were employed. Initially, Coulombic solute–solvent interactions were disabled at higher λ values compared to *vdW* terms to prevent unstable Coulombic interactions, which could yield unreliable energies and configurations^46,49^. Soft-core potentials with specific parameters (α = 0.5, σ = 0.3, and *p* = 1) were integrated to prevent atom overlap between solute and solvent at low λ values^46–48^. Free energy calculations commenced from the final configuration of each simulated system, with each simulation lasting 5 ns.

## Results and discussion

### Interaction mechanism

**Figure 3:**
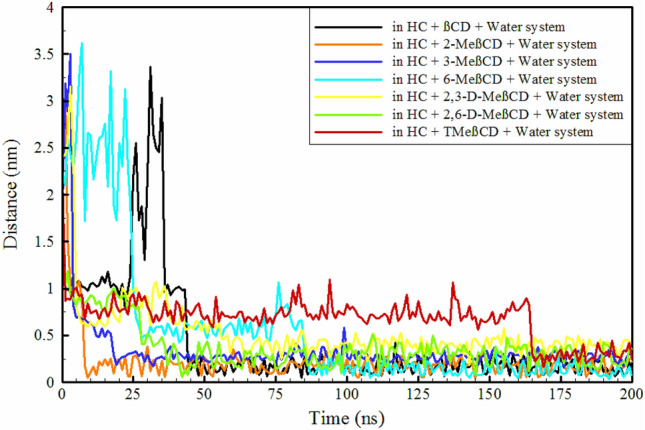
Time evaluation the distance between Hydrocortisone and the center of CD molecules.

CDs enhance the stability of pharmaceutical substances by protecting them from degradation, oxidation, or hydrolysis^6,50^. This protective effect is facilitated by the encapsulation of drugs within the cavity of CDs, effectively isolating them from external factors that could compromise their effectiveness or durability^7,51^. The interaction mechanism between CDs and pharmaceutical compounds is analyzed through the formation of inclusion complexes. This process leads to improvements in solubility, stability, release kinetics, and bioavailability, thereby enhancing the therapeutic effectiveness of the pharmaceutical agent^52–55^. The initial and final configurations of all HC-containing systems are illustrated in Fig. 2. The cavities of all CDs show the ability to host HC. Among these, HC is positioned in βCD, 2-MeβCD, 3-MeβCD, 6-MeβCD and TMeβCD with the hydroxyl groups facing the PHR and the ketone side (oxygen group attached to the aromatic ring) facing the SHR. It appears that the loading of HC into the cavities of βCD and 6-MeβCD is more complete, as a larger portion of the HC molecule is accommodated in their cavities. In contrast, loading of HC into the cavities of 2,3-D-MeβCD and 2,6-D-MeβCD follows a different mechanism, with the ketone side facing PHR and the hydroxyl groups facing SHR. In addition, it appears that loading into the cavity of 2,3-D-MeβCD is more complete than in 2,6-D-MeβCD. Although the final positioning of HC is clearly defined, understanding the time evolution of HC entry into the CDs cavities can provide insight into the kinetics of these interactions. To this end, the distances between the centre of geometry (COG) of the HC and the CDs are depicted in Fig. 3. As seen, loading of HC into the cavities of βCD, 6-MeβCD, 2,3-D-MeβCD, 2,6-D-MeβCD and TMeβCD occurs gradually, with entry times of approximately 43.6, 84.1, 55.1, 30.6 and 164.7 ns, respectively. In contrast, entry into the cavities of 2-MeβCD and 3-MeβCD is faster, with entry times of approximately 7.4 and 17.1 ns, respectively. Figure S2 illustrates the moment and mode of HC entry into the CDs cavities. It is evident that in βCD and 6-MeβCD, HC approaches the PHR from its ketone side, so that the efficient loading is facilitated. Conversely, in 2-MeβCD, 3-MeβCD, and TMeβCD, HC approaches the SHR from its hydroxyl groups side, leading to loading. Finally, in 2,3-D-MeβCD and 2,6-D-MeβCD, entry of HC occurs from the ketone side towards the SHR.

### Root mean square fluctuation (RMSF)

**Figure 4:**
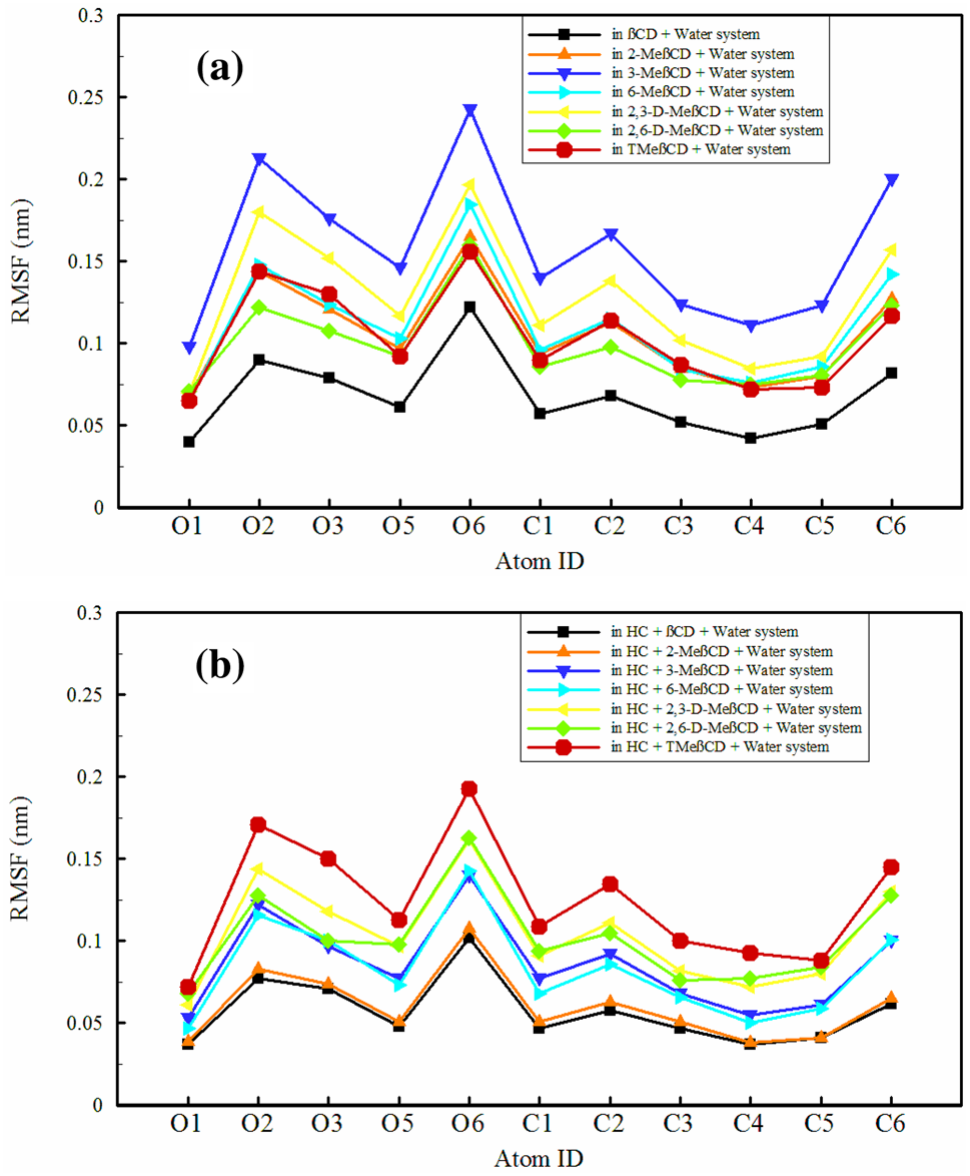
Root mean square fluctuation (RMSF) of heavy atoms of CDs in different simulated system.

RMSF is crucial in MD simulations for understanding molecule flexibility, such as CDs in drug delivery. It reveals atom fluctuation, helping identify flexible or rigid parts. CD flexibility affects guest molecule binding, with RMSF analysis pinpointing conformational changes. RMSF also predicts encapsulation efficiency, vital for optimizing CD-based systems, improving drug carrier performance^48,56–58^. Here, we first consider the changes in the flexibility of the heavy atoms forming the main skeleton in all methylated derivatives as well as in βCD, followed by a discussion of the changes induced by the presence of HC. As shown in Fig. 4a, among the oxygen atoms in all structures, O6 shows the highest flexibility. This oxygen atom, located at the end of a short chain and distant from the ring, exhibits greater degrees of freedom and flexibility. Conversely, O1 and O5 show the least flexibility, probably due to their position within the solid ring structure. In terms of carbon atoms, C6 shows the highest flexibility, while the carbon atoms within the ring (C1, C3, C4, C5) show much less flexibility. After O6 and C6, the highest flexibility of all oxygen and carbon atoms is observed for O2 and C2. It seems that these two atoms within the ring have greater flexibility compared to others. As a result, methylation increases the flexibility of all heavy atoms, with 3-MeβCD and 2,3-D-MeβCD exhibiting the highest flexibility, distinguishing them from other structures. Remarkably, in the presence of HC (as shown in Fig. 4b), the heavy atoms within 3-MeβCD and 2,3-D-MeβCD, as well as 2-MeβCD and 6-MeβCD, undergo a significant reduction in flexibility. This observation suggests that these methylated derivatives have a more rigid structure compared to their counterparts. It appears that upon inclusion of HC, these four structures lose their flexibility and effectively retain HC within their cavities. Meanwhile, βCD exhibits the least flexibility as the parent structure in the absence of HC. The changes in the RMSF of its heavy atoms are not as severe in the presence of HC, and it remains as the most rigid structure.

### Water penetration into the cavity

**Figure 5:**
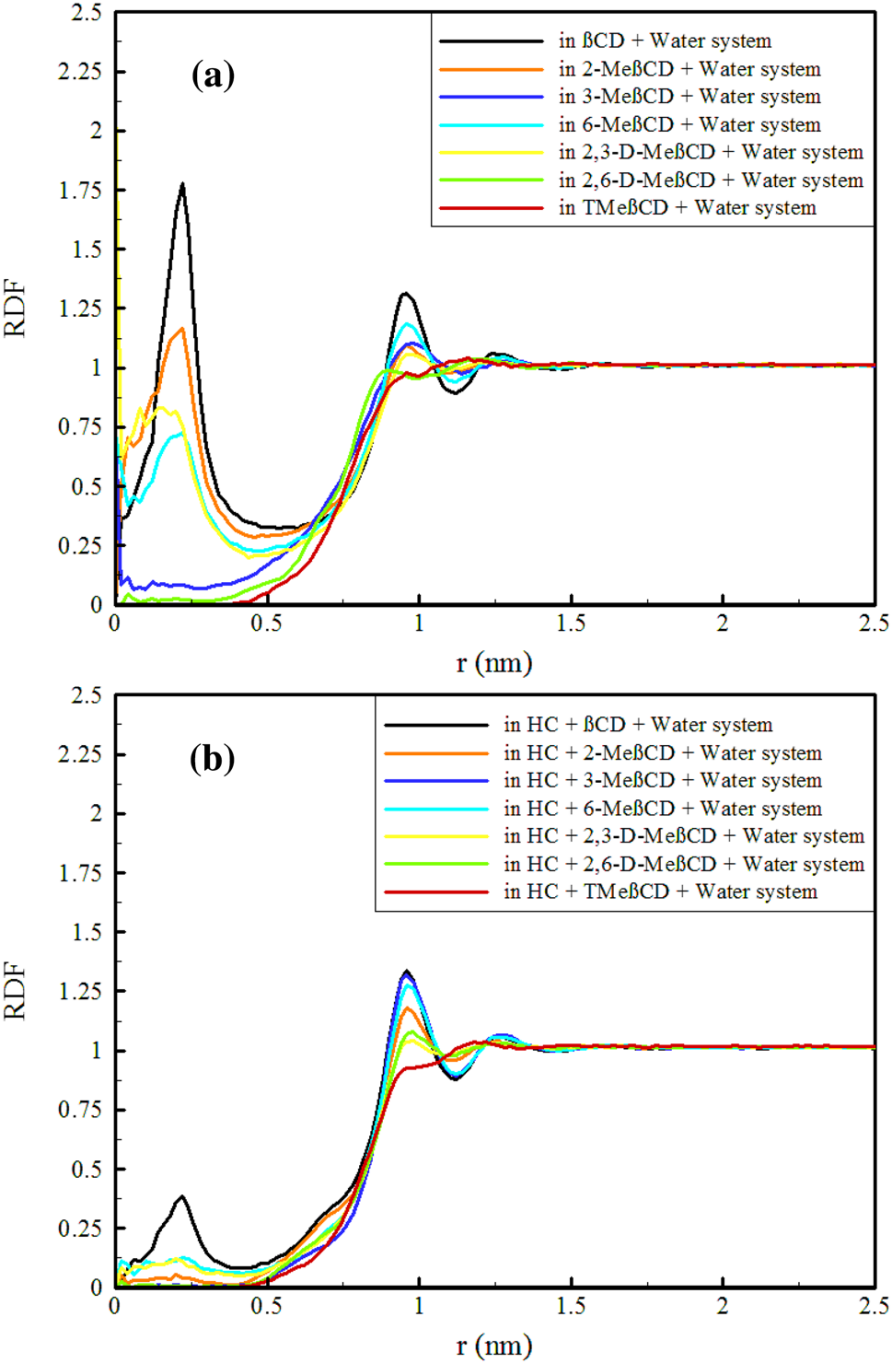
Radial distribution function (RDF) of water around CDs in different simulated systems.

In the absence of a guest molecule, it is common for water molecules to typically fill the CD cavity within an aqueous solution. X-ray and neutron diffraction analyses reveal that α-CD, β-CD, and γ-CD cavities typically contain 2.6, 6.5, and 8.8 water molecules, respectively, distributed within different layer of the cavities^59–64^. These confined water molecules exist in a high-energy state due to the lack of a complete hydrogen bond network. However, upon the introduction of a guest molecule into the cavity, these high-energy water molecules are released, facilitating the formation of a stable cavity-guest complex. This phenomenon indicates the thermodynamic favorability of cavity-guest complexation within CD cavities in aqueous solutions^65–68^. Hence, the probability of the presence of water molecules and their arrangement around CDs was determined using the Radial Distribution Function (RDF), as depicted in Fig. 5.

As observed, βCD contains a significant number of water molecules at distances of less than 0.5 nm (the innermost layer). In this layer, the probability of the presence of water in the methylated derivatives is reduced, with a more pronounced reduction observed for 3-MeβCD, 2,6-D-MeβCD and TMeβCD, while the outflow of water is less pronounced for 2-MeβCD, 2,3-D-MeβCD and 6-MeβCD, with significant amounts of water still remaining. For a quantitative assessment, considering the location of the first minimum in the RDF at 0.5 nm, hypothetical spheres with different radii were considered in the central region of the CDs cavities and the number of water molecules present in these spheres was counted, as shown in Table S2. The average number of water molecules in this layer (0–0.5 nm) for βCD is 9.9, which is in good agreement with other simulation studies.

As a result of functionalization, there was a significant reduction in the number of water molecules in the innermost layer (0–0.5). The reduction in the number of water molecules in this layer ranges from a minimum of 42% (as observed for 2-MeβCD, with 5.74 water molecules) to a maximum of 97% (as observed for TMeβCD, with 0.25 water molecules). This can be attributed to the decrease in the polarity inside the cavity. Significant changes also occurred in the outermost layer (0.5–1.0), with reductions ranging from a minimum of 1.3% (as observed for 6-MeβCD, with 88.88 water molecules) to 15.4% (as observed for TMeβCD, with 76.15 water molecules). Upon HC loading, a significant release of high-energy water molecules from the innermost layer (0–0.5) of all CD cavities is observed, creating more favorable conditions for HC loading into the cavity. The second layer (0.5–1.0) also experienced a decrease in the number of water molecules due to the presence of HC. Although this decrease was noticeable, it was not as great as in the innermost layer.

### Conformational changes

Understanding CDs conformational changes is vital for enhancing drug delivery systems, optimizing material properties, and designing molecular recognition systems. It influences host–guest interactions, nanostructure characteristics, and impacts stability, toxicity, and biological interactions. This knowledge is instrumental in creating advanced drug formulations and functional materials for diverse applications^69–71^. The most significant structural changes in various simulated systems of CDs have been summarized in Table 1. Instructions on computing both the area and volume of the cavity were provided in the Supplementary Information (**page 4**), and these methods have been confirmed in other simulation studies^72,73^. The functionalization of βCD and the subsequent formation of methyl derivatives leads to a partial decrease of the A_PHR_ in all methylated derivatives, with a notable reduction observed mainly in 2,3-D-MeβCD (1.00 nm^2^) and to a lesser extent in 3-MeβCD (1.02 nm^2^). The presence of HC increases the A_PHR_ in almost all derivatives. Particularly, for 2,6-D-MeβCD (1.28 nm^2^) and 6-MeβCD (1.27 nm^2^), a more pronounced increase is observed. This phenomenon can be attributed to the functionalization of position **R**_**6**_ in both structures, rendering this region more hydrophobic. The repulsive interaction between the hydrophilic oxygen of HC and these hydrophobic groups contributes to the increase in A_PHR_. Conversely, the significant decrease in A_PHR_ observed in 2,3-D-MeβCD (0.97 nm^2^) can be explained by the mechanism of HC loading into its cavity. The attractive interaction between the hydroxyl groups present in this rim and the oxygen of HC, which penetrates deep into the cavity, leads to a significant decrease in A_PHR_. The changes in A_MID_ in methylated derivatives are much more severe, showing a significant decrease compared to βCD (0.83 nm^2^). It appears that methylation causes the internal part of the cavities of the derivatives to become highly non-polar, leading to intramolecular attraction in this region. Such an event has previously been observed in some βCD derivatives, accompanied by a significant outflow of water from the center of the cavity^46^. However, the loading of HC into the cavities leads to an increase in the A_MID_ values, which are almost the same for all derivatives as for βCD. As can be seen, the changes in A_SHR_ are also very significant in the methylated derivatives. The largest decrease is observed in 2,6-D-MeβCD, while the smallest decrease is observed in 2,3-D-MeβCD. It seems that the presence of two adjacent methyl groups at positions **R**_**2**_ and **R**_**3**_ causes some repulsion and prevents a further decrease in A_SHR_. However, upon HC loading, A_SHR_ increases significantly in all derivatives, whereas it decreases in βCD. It seems that for HC loading, A_SHR_ shows much greater structural desirability in methylated derivatives compared to βCD. In both the presence and absence of HC, βCD exhibits high circularity (Ω_O1_ ≈ 0.99). Despite the limited degree of freedom of the O1 atoms due to bonding with carbon atoms (C1 and C4) within the ring, functionalization of βCD and the formation of methyl structures lead to a decrease in Ω, especially notable in 3-MeβCD (0.92). Upon loading HC, increased steric repulsion interactions between its bulky atoms and the O1 atoms of the carriers appear to result in a more regular and nearly equidistant equilibrium position of the O1 atoms with respect to each other. Consequently, this increases Ω_O1_ across all carriers, yielding very similar Ω_O1_ values among them. The CDs heights (*h*_*12*_ and *h*_*16*_) also experience a significant reduction. Specifically, the values of 0.33 (*h*_*16*_) and 0.22 (*h*_*12*_) nm in βCD decreased to 0.22 and 0.12 nm in 2,6-D-MeβCD, and to 0.21 and 0.13 nm in TMeβCD. Other methylated derivatives also experience significant changes in this area, as shown in Table 1. Considering the defined equation for the determination of the volume of CDs cavities (page 4 of the SI), it is clear that what influences the volume of the cavities (*V*_*C*_) more than other structural changes is precisely the height and not the surfaces. Methylated derivatives of βCD seem to undergo substantial height changes after HC loading and are drawn along the $\mathop{r}\limits^{\rightharpoonup}$ axis (Fig. 1). This consequently leads to a significant increase in *V*_*C*_, with the largest observed in 6-MeβCD and 2,6-D-MeβCD at 0.61 nm^3^ and 0.60 nm^3^, respectively. The reduction in *V*_*C*_ of the methylated derivatives in comparison with βCD can be attributed to the increase in the non-polar nature of these cavities.

**Table 1:** Conformational Parameters Describing Molecular Arrangement of CDs in different simulated systems.

Systems	Structural Properties						
	*A*_PHR_ (nm^2^)	*A*_MID_ (nm^2^)	*A*_SHR_ (nm^2^)	Ω_O1_	*h*_12_ (nm)	*h*_16_ (nm)	*V*_C_ (nm^3^)
Water + βCD	1.10	0.83	1.32	0.99	0.22	0.33	0.55
Water + βCD + Hydrocortisone	1.14	0.84	1.28	0.99	0.22	0.33	0.56
Water + 2-MeβCD	1.05	0.79	1.20	0.96	0.18	0.27	0.42
Water + 2-MeβCD + Hydrocortisone	1.16	0.85	1.30	0.99	0.23	0.33	0.57
Water + 3-MeβCD	1.02	0.72	1.16	0.92	0.15	0.24	0.35
Water + 3-MeβCD + Hydrocortisone	1.11	0.83	1.37	0.97	0.21	0.33	0.55
Water + 6-MeβCD	1.04	0.76	1.16	0.94	0.17	0.27	0.40
Water + 6-MeβCD + Hydrocortisone	1.27	0.84	1.29	0.97	0.23	0.35	0.61
Water + 2,3-D-MeβCD	1.00	0.80	1.25	0.97	0.19	0.29	0.45
Water + 2,3-D-MeβCD + Hydrocortisone	0.97	0.83	1.39	0.98	0.20	0.33	0.52
Water + 2,6-D-MeβCD	1.05	0.70	1.08	0.95	0.12	0.22	0.30
Water + 2,6-D-MeβCD + Hydrocortisone	1.28	0.85	1.31	0.97	0.22	0.34	0.60
Water + TMeβCD	1.06	0.72	1.12	0.96	0.13	0.21	0.30
Water + TMeβCD + Hydrocortisone	1.19	0.85	1.39	0.98	0.21	0.34	0.58

*A*_PHR_, *A*_MID_, and *A*_SHR_ are the areas of primary hydroxyl rim, middle rim, an secondary hydroxyl rim (the rings that are formed by connecting the O6, O1, and O3 atoms, respectively); *h*_1j_ is the distance between the center of mass of O1 atoms and the center of mass of Oj atoms; Ω_O1_ is the circularity of rim comprising O1 atoms defined as the ratio of the smallest to the largest distance between any pair of O1 atoms in the rim; CDs height, *h*, is the distance between the centers of mass of the primary and the secondary hydroxyl rims (*h* = *h*_12_ + *h*_16_); Vc is the volume of the cavity for CDs in different simulated systems. All results were obtained from the last 10% of the simulation time.

### Relative shape anisotropy and surface-to-volume ratio

Relative shape anisotropy (RSA), also known as *κ*^*2*^, refers to the deviation of the shape of a polymer molecule from that of a perfect sphere or cylinder^74,75^. This characteristic is critical in drug delivery, as it affects the distribution and retention of drug carriers in the body, as well as their transport across biological barriers such as cell membranes and tissue extracellular matrix. Understanding the RSA of drug carriers is essential for improving the efficacy of drug delivery and minimizing side effects^76,77^. The calculation of RSA involves utilizing the principal moments of the radius of gyration (Rg), typically arranged as λ_1_ ≥ λ_2_ ≥ λ_3_, and summing them to obtain ${\text{R}}_{\text{g}}^{2}={\uplambda }_{1}+{\uplambda }_{2}+ {\uplambda }_{3}$, from which RSA can be derived^78^:

2 $$\text{RSA}=1-3\frac{({\lambda }_{1}{\lambda }_{2}+{\lambda }_{2}{\lambda }_{3}+{\lambda }_{1}{\lambda }_{3})}{{({\lambda }_{1}+{\lambda }_{2}+{\lambda }_{3})}^{2}}$$

**Figure 6:**
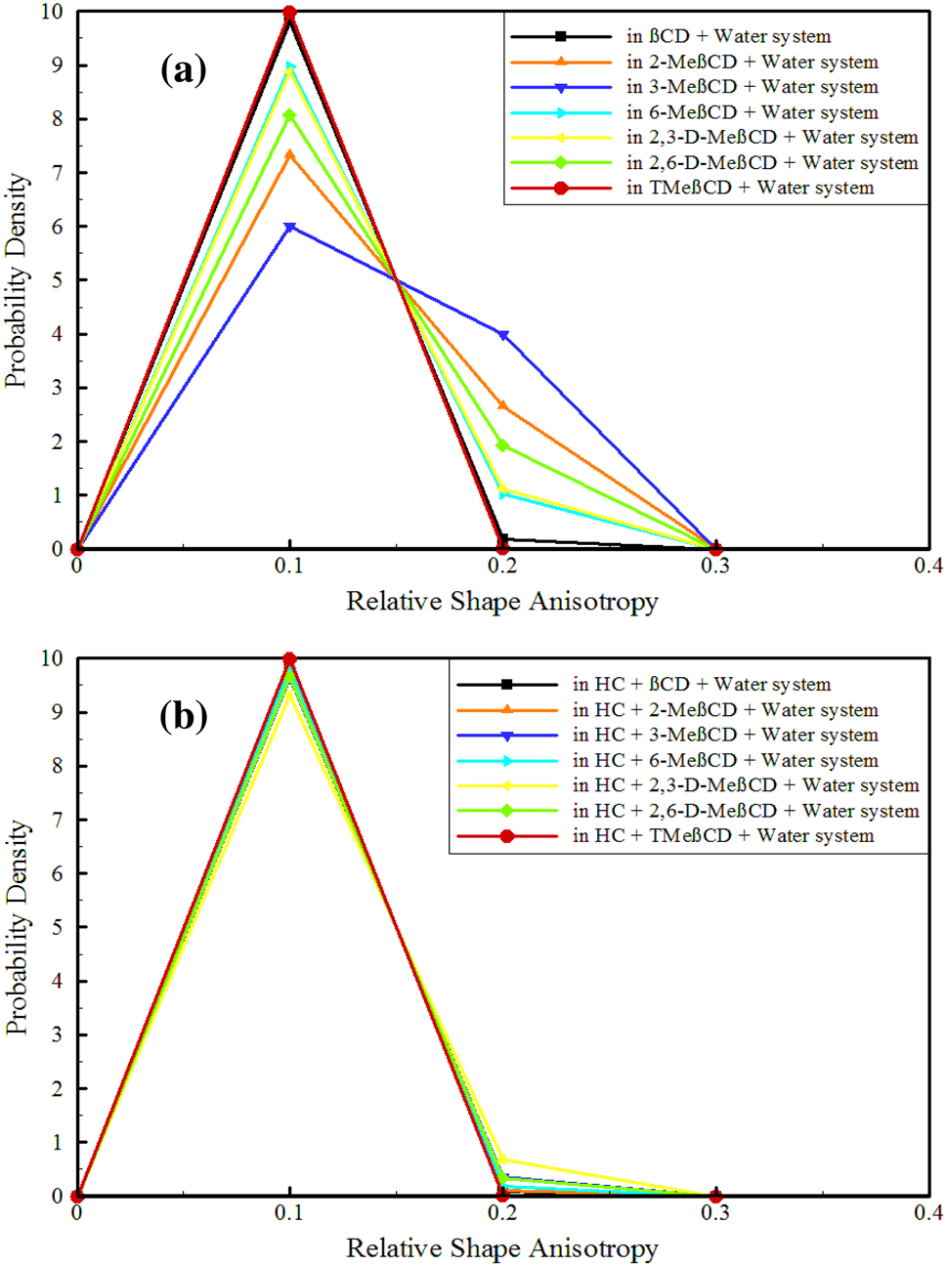
The relative shape anisotropy parameter of CDs in different simulated systems.

RSA can take values between 0 and 1, with a value of 0 corresponding to a highly symmetric polymer conformation and reaching 1 for an ideal linear chain. The examination of RSA for all CDs with and without HC was carried out, as shown in Fig. 6. In the absence of HC, βCD and TMeβCD demonstrated the highest symmetry, with RSA showing the highest probability around 0.1 (Fig. 6a). However, due to functionalization, all other methylated derivatives exhibited noticeable asymmetry compared to βCD, with a significant probability concentrated around RSA = 0.2. The methylated derivatives 3-MeβCD, 2-MeβCD, and 2,6-D-MeβCD displayed the greatest level of asymmetry, with probabilities around 4, 3, and 2, respectively. This points to a significant departure from symmetry for these methylated derivatives. Loading the HC altered everything (Fig. 6b), causing all CDs to have highly symmetric structures with the highest probability near RSA = 0.1. Such that a significant probability is not observed around RSA = 0.2, means that the symmetry increases sharply. Only in the case of 2,3-D-MeβCD, to some extent, a probability (~ 0.8) is still observable around RSA = 0.2.

**Figure 7:**
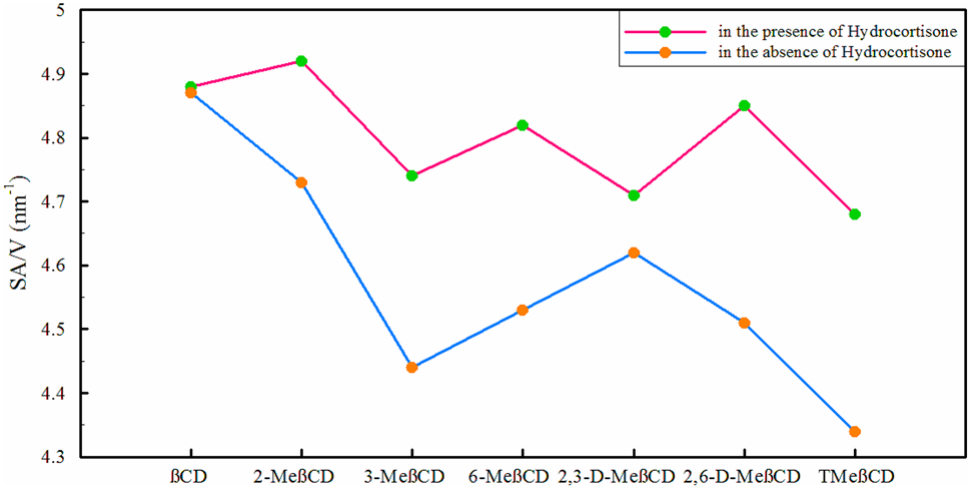
The surface-to-volume ratio (SA/V) in different simulated systems.

The findings suggest that elongating CDs along the $\mathop{r}\limits^{\rightharpoonup}$ axis (as illustrated in Fig. 1 and Table 1) leads to a more symmetrical structure in HC-containing systems. This symmetrical configuration may not only enhance dissolution and drug delivery but also offers additional benefits. The larger surface area of nanoparticles relative to their volume facilitates increased interaction with the environment, thereby enabling various applications such as drug delivery and catalysis^79,80^. Hence, we assessed the ratio of the solvent-accessible surface area (SASA) of each CD to its volume, as shown in Fig. 7.

As can be seen, in the presence of HC, the SA/V ratio of all CDs increases. The lowest changes correspond to βCD, followed by 2,3-D-MeβCD, and 2-MeβCD. The changes appear to be height-dependent. After functionalization and HC loading, 2,3-D-MeβCD and 2-MeβCD exhibit minimal height (*h*_*12*_ + *h*_*16*_) alterations in comparison to βCD, as shown in Table 1. Conversely, 3-MeβCD, 6-MeβCD, 2,6-D-MeβCD, and TMeβCD experience significant height changes following methylation and HC loading. Overall, the highest SA/V ratio in the presence of HC is associated with 2-MeβCD, βCD, and 2,6-D-MeβCD, respectively. It's important to note, however, that the circular polymer structure of CDs limits the potential expansion of surface area.

### Analysis of interaction energies and binding free energy

The interaction energies between the solute and solvent determine the solubility and stability of a compound in a solution. These energies depend largely on Coulombic forces, vdW forces, and hydrogen bonding interactions. The magnitude and orientation of these interactions are affected by the chemical characteristics and shape of both the solute and solvent molecules. Understanding these interactions is essential for designing novel carriers, improving the conditions for reactions, and synthesizing new materials^81–83^.

**Figure 8:**
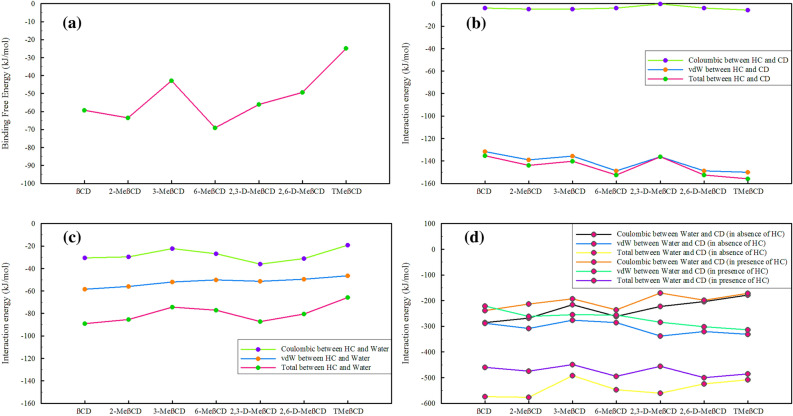
Energy Analysis among Various Components within Simulated Systems. (All results were obtained from the last 10% of the simulation time.).

The BAR method was employed to determine the solvation free energy (ΔG_solv_) and binding free energy (ΔG_bind_) of HC to CDs, as described previously. The ΔG_solv_ of HC in water was found to be somewhat negative (-13.91 kJ/mol), as expected due to the abundance of hydroxyl groups, despite the hydrophobic nature of the main skeleton. It is evident that the binding of HC to all CDs was spontaneous and energetically favorable. Among these, the most exothermic processes, in order, are associated with the binding of HC to 6-MeβCD, 2-MeβCD, βCD, 2,3-D-MeβCD, 2,6-D-MeβCD, 3-MeβCD, and TMeβCD, as seen in Fig. 8a. Further insights into the forces involved in binding and solvation were gained by analyzing various types of interaction energies in simulated systems.

Simultaneous investigation was conducted to examine the energy interactions between different components in various systems, as depicted in Fig. 8. The evidence suggests that in the interaction between HC and CDs, the *vdW* forces predominantly drive the thermodynamic favourability, while the Coulombic interactions have a minor effect (Fig. 8b). Among these interactions, HC showed more thermodynamic favorability with 6-MeβCD, 2,6-D-MeβCD and TMeβCD derivatives compared to other CDs, as indicated by more negative energy values. An interesting observation is that the thermodynamic interaction of HC with methylated derivatives appears to be more favourable than its interaction with βCD. This underlines the potential of these methylated derivatives as promising candidate carriers for HC. In the water/HC system, the energies of the *vdW* and Coulombic interactions between HC and water were measured to be -118.53 and -47.13 kJ/mol, respectively. In the presence of CDs, the contributions of Coulombic and *vdW* interactions are significantly reduced (Fig. 8c), ensuring that in none of the systems do the Coulombic and *vdW* interaction energies between HC and water (total energy) exceed the *vdW* interaction energy of HC and CDs. Consequently, the loading of HC in all CDs is assessed as thermodynamically favorable. Another important interaction that can determine the solubility of the inclusion complex is the interaction of CDs with water in the presence and absence of HC, Fig. 8d. As a result of methylation, the contribution of the Coulombic interaction between water and CDs is lower in all methylated derivatives than in βCD (energies become more positive), and a Coulombic repulsion due to methylation is observed. Conversely, the contribution of the vdW interaction remains almost unchanged (as seen in 3-MeβCD and 6-MeβCD) or increases in thermodynamic favorability (as seen in 2-MeβCD, 2,3-D-MeβCD, 2,6-D-MeβCD, TMeβCD), which is manifested by the negative shift of the energy of the vdW interaction between CDs and water. In the presence of HC, the Coulombic repulsion in 2,6-D-MeβCD and TMeβCD remains almost unchanged, while in 3-MeβCD and 6-MeβCD, it is partially accompanied by a positive shift. However, it can be seen that a Coulombic repulsion between the CDs and water occurs due to the presence of HC in βCD, 2-MeβCD, and 2,3-D-MeβCD. The same pattern and effect also occur for the contribution of the vdW interaction energies between CDs and water in the presence of HC. However, an interesting observation regarding the total interaction energies between CDs and water in the presence of HC shows that the energy of interaction of methylated derivatives with water in the presence of HC is more negative (in 2-MeβCD, 6-MeβCD, 2,6-D-MeβCD, TMeβCD) or almost equal to βCD (in 3-MeβCD and 2,3-D-MeβCD) as the parent structure, providing further evidence for the thermodynamic favorability of methylated structures as carriers of HC.

### Hydrogen bonds

The significance of hydrogen bonding is essential in "host–guest" systems, as it is crucial for selective binding, stability, shape recognition, control of properties, reversibility, and can impact the pharmacokinetics of drugs^84,85^. Hence, the assessment of hydrogen bond formation ability among different components in simulation systems was carried out, with findings detailed in Table 2. HC in water (in the absence of any CD) forms 31.8 hydrogen bonds with water. Loading HC into CDs significantly reduces the number of this type of hydrogen bonds, with the greatest reduction resulting from loading into 3-MeβCD (1.11) and TMeβCD (0.97). The reason for this phenomenon could be elucidated by considering the mechanism of HC loading. In 3-MeβCD and TMeβCD, as discussed earlier, the loading of HC occurs in such a way that the hydroxyl groups of HC are completely covered by the cavity of the CDs (Fig. 2), making them inaccessible to water. As a result, the number of hydrogen bonds between HC and water is greatly reduced. In 2,3-D-MeβCD (2.37) and 2,6-D-MeβCD (1.96) the highest number of hydrogen bonds between HC and water is observed compared to the other derivatives. It is also evident that due to the loading mechanism of HC into the cavities of 2,3-D-MeβCD and 2,6-D-MeβCD, where only the ketone side of HC is completely covered within the cavities, the hydroxyl groups of HC are fully accessible to water molecules to form hydrogen bonds. Finally, the quality and persistence of hydrogen bonding (as indicated by the lifetime in ps) between HC and water is almost identical in all systems (~ 11 ps). A likely conclusion from these observations is that a higher number of hydrogen bonds between HC and water may lead to a faster release process. The number of hydrogen bonds between CDs and water also indicates that βCD is more capable of forming hydrogen bonds with water (19.41) than other CDs. The formation of methylated derivatives leads to a significant decrease in the number of hydrogen bonds. Among these, the most significant reduction is attributed to TMeβCD (9.73), while the least reduction is associated with 2-MeβCD (16.42) and 6-MeβCD (16.56). Therefore, methylation is considered thermodynamically undesirable for the solubility of CDs, although it is not the only thermodynamic determinant. HC forms hydrogen bonds with all CDs almost equally and imperceptibly, which certainly cannot be considered as the determining factor for HC loading. However, the most important observation is the significant decrease in the number of intramolecular hydrogen bonds of methylated derivatives compared to βCD. The presence of a full intramolecular hydrogen bond belt in βCD makes it rigid (between hydroxyl groups at O_2_ and O_3_ position), leading to lower water solubility than α-CD and γ-CD^86^. As observed, methylation leads to a significant disruption of the intramolecular hydrogen bonding network, resulting in a noticeable reduction in the number of intramolecular hydrogen bonds in methylated derivatives compared to βCD (5.94). The highest reduction is associated with TMeβCD (0.00) and the lowest reduction with 2-MeβCD (4.10). This observation can be thermodynamically evaluated as favorable for enhancing the solubility of CDs. It appears that the loading of HC increases this type of hydrogen bonding, which in turn reinforces the mentioned network and leads to the stiffening of the methylated derivatives, as evident in the "RMSF" (as discussed in Sect. "Root mean square fluctuation (RMSF)").

**Table 2:** Average number of different hydrogen bonds and Life-Time (ps) in the simulated systems.

System	H-bond			
	Between water and Hydrocortisone (Life-Time)	Between water and CD (Life-Time)	Between Hydrocortisone and CD (Life-Time)	Between CD and CD (Life-Time)
Water + βCD	–	19.41 (10.69)	–	5.94 (29.02)
Water + βCD + Hydrocortisone	1.83 (10.65)	15.47 (10.85)	0.71 (11.82)	7.46 (35.65)
Water + 2-MeβCD	–	16.42 (11.61)	–	4.10 (22.04)
Water + 2-MeβCD + Hydrocortisone	1.82 (10.80)	12.27 (11.70)	0.61 (12.57)	7.27 (37.65)
Water + 3-MeβCD	–	14.50 (11.32)	–	2.10 (15.43)
Water + 3-MeβCD + Hydrocortisone	1.11 (10.57)	11.90 (10.69)	0.80 (12.26)	4.76 (22.90)
Water + 6-MeβCD	–	16.56 (11.10)	–	3.18 (3080)
Water + 6-MeβCD + Hydrocortisone	1.62 (10.85)	14.08 (11.17)	0.68 (15.14)	4.86 (55.89)
Water + 2,3-D-MeβCD	–	14.35 (12.56)	–	1.31 (14.30)
Water + 2,3-D-MeβCD + Hydrocortisone	2.37 (10.84)	10.55 (12.17)	033 (12.82)	2.10 (16.36)
Water + 2,6-D-MeβCD	–	11.40 (11.20)	–	2.14 (29.05)
Water + 2,6-D-MeβCD + Hydrocortisone	1.96 (11.02)	10.76 (11.83)	0.41 (14.17)	4.19 (42.20)
Water + TMeβCD	–	9.73 (13.31)	–	0.00
Water + TMeβCD + Hydrocortisone	0.97 (10.74)	9.52 (14.33)	0.56 (12.64)	0.00

All results were obtained from the last 10% of the simulation time.

## Conclusion

This comprehensive computational study has provided a detailed understanding of the interactions between hydrocortisone and various CD derivatives, including methylated βCDs. The results demonstrate the versatility of CDs as drug delivery carriers and highlight the importance of molecular-level investigations in optimizing these systems. The interaction mechanism between HC and the CDs was elucidated through the formation of inclusion complexes, which led to improvements in solubility, stability, and loading kinetics. The specific positioning and orientation of HC within the CD cavities were found to be crucial, with the hydroxyl groups of HC facing the primary hydroxyl rim and the ketone side facing the secondary hydroxyl rim in most cases. The kinetics of HC entry into the CD cavities varied significantly, with some derivatives exhibiting faster loading times compared to others. The analysis of structural flexibility, as measured by root mean square fluctuation (RMSF), revealed that methylation enhances the flexibility of the CD structures, but the presence of HC can lead to a reduction in flexibility, particularly for 3-MeβCD, 2,3-D-MeβCD, 2-MeβCD, and 6-MeβCD. This suggests that these derivatives may be more effective in retaining the guest molecule within their cavities. The investigation of water penetration into the CD cavities provided insights into the thermodynamic favorability of the cavity-guest complexation. The reduction in the number of high-energy water molecules within the innermost layer of the methylated CDs upon HC loading indicates the release of these water molecules, facilitating the formation of a stable inclusion complex. The conformational changes observed in the CD structures, such as the changes in the areas of the primary, middle, and secondary hydroxyl rims, as well as the cavity volume and circularity, further highlight the impact of functionalization and guest loading on the structural properties of these carriers. These changes can directly influence the solubility, stability, and drug release characteristics of the CD-based formulations. Overall, this study underscores the potential of CDs, particularly methylated derivatives, as effective drug delivery platforms for hydrophobic compounds like hydrocortisone. The insights gained from the computational analysis can guide the rational design and optimization of CD-based pharmaceutical formulations to enhance the therapeutic efficacy and bioavailability of challenging drug candidates.

### Supplementary Information

Supplementary Information.

## Data Availability

The datasets used and/or analyzed during the current study are available from the corresponding author on reasonable request.
