# Supplementary material for: Unveiling the dynamic and thermodynamic interactions of hydrocortisone with β-cyclodextrin and its methylated derivatives through insights from molecular dynamics simulations
Source: Sci Rep. 2024 May 31;14:12495. doi: 10.1038/s41598-024-63034-7 (PMC11143220; doi:10.1038/s41598-024-63034-7)
Supplement: Supplementary file 1 — Supplementary Information. [file 41598_2024_63034_MOESM1_ESM.pdf]

Supplementary Information: Unveiling the Dynamic and Thermodynamic Interactions of Hydrocortisone with β-Cyclodextrin and Its Methylated Derivatives through Insights from Molecular Dynamics Simulations

Roya Gholami[a], Khaled Azizi[a,b,], Mokhtar Ganjali Koli [a,b]*

[a]*Department of Chemistry, University of Kurdistan, Sanandaj, Iran*

[b]*Computational Chemistry Laboratory, Kask Afrand Exire Ltd., Sanandaj, Iran*

*** Corresponding Author:**

Email: k.azizi@uok.ac.ir

Contents

Figure S1: Nomenclature and definitions used in this article for structural parameters of β-Cyclodextrin…………………..……..………………………………………………………………**3**

Table S1: Structural Properties of β-Cyclodextrin obtained from this MD Simulations and Experiment…………………..……………………………..…………………………….….….**3**

Figure S2: Snapshots of the moment and mechanism of Hydrocortisone entry into the CDs cavities……………………...……………………..…………………………………………….**4**

Calculation methods for area and volume of cavity……..….……..……………….………..…..**5**

Table S2: Number of water molecules in different spheres inside the CDs cavity…..…..….….**6**

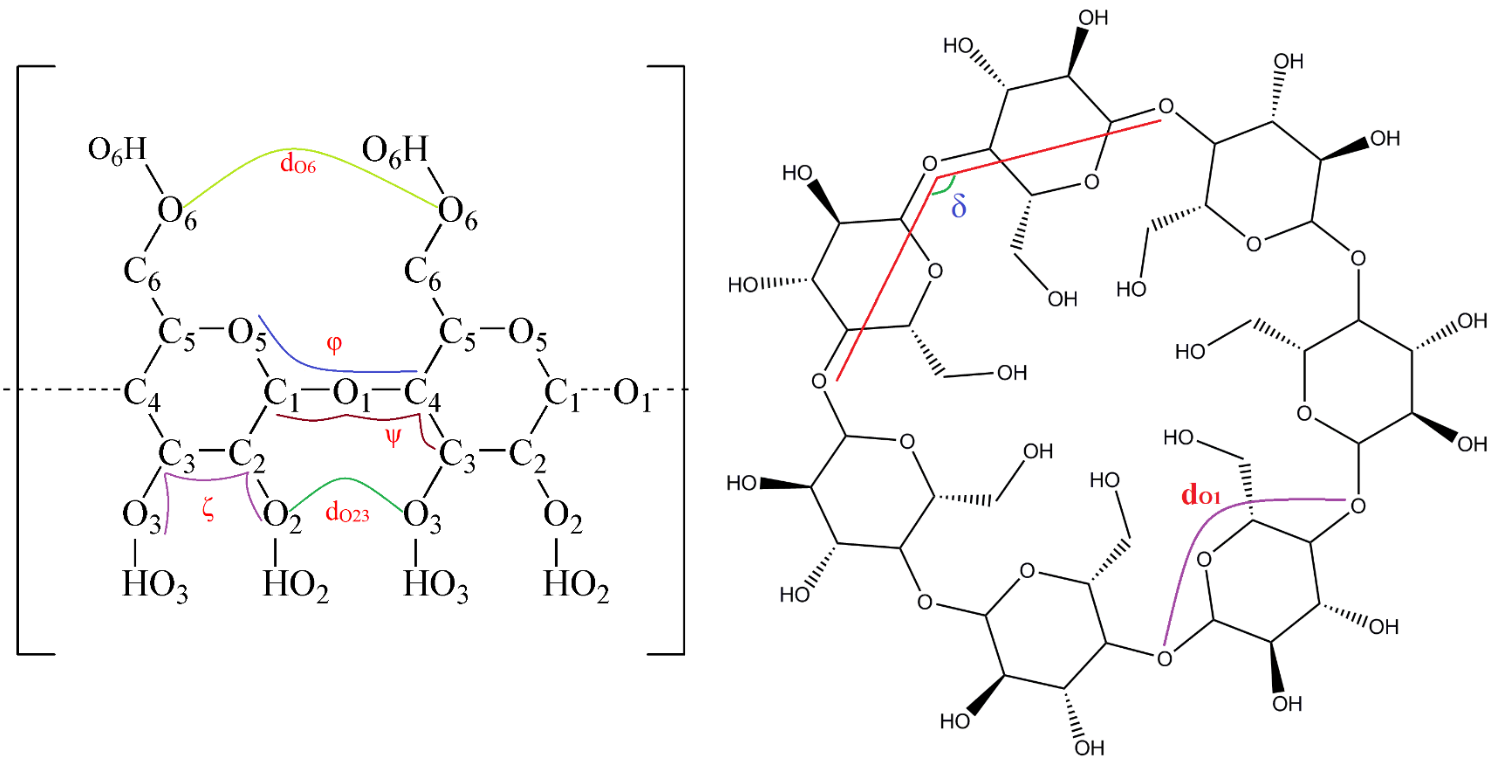

Figure S1: Nomenclature and definitions used in this article for structural parameters of β-Cyclodextrin.

Table S1: Structural Properties of β-Cyclodextrin obtained from this MD Simulations and Experiment[a].

	φ dihedral angle [°]	ψ dihedral angle [°]	ζ dihedral angle [°]	δ angle [°]	d_{O1} [nm]	d_{O6} [nm]	d_{O23} [nm]	Circularity[b]	Radius of gyration [nm]	1st rim diameter (nm)	2nd rim diameter (nm)
Experimental	109.8[c]	127.6[c]	63.2[d]	128.3[c]	0.4385[c]	0.5384[d]	0.2884[c]	0.94[e]	0.60[f]	1.01[f]	1.25[f]
This work	121.43	116.84	59.54	127.63	0.4488	0.4528	0.2990	0.98	0.60	0.996	1.29

[a]All results were obtained from the last 10 % of the simulation time. [b]ratio of the smallest to the largest distance between any pair of glucose O1 atoms that lie across the ring from each other. [c]Reference [1], [d]Reference [2], [e]Reference [3], [f]Reference [4]

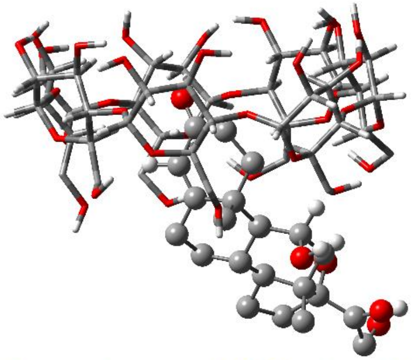

Hydrocortisone and **βCD** after 43.6 ns

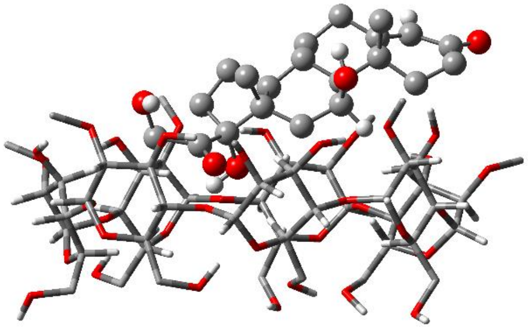

Hydrocortisone and **2-MeβCD** after 7.4 ns

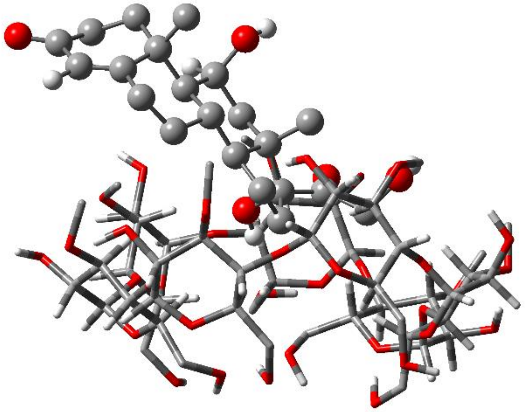

Hydrocortisone and **3-MeβCD** after 17.1 ns

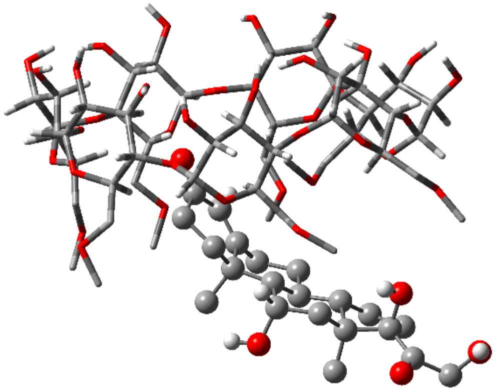

Hydrocortisone and **6-MeβCD** after 84.1 ns

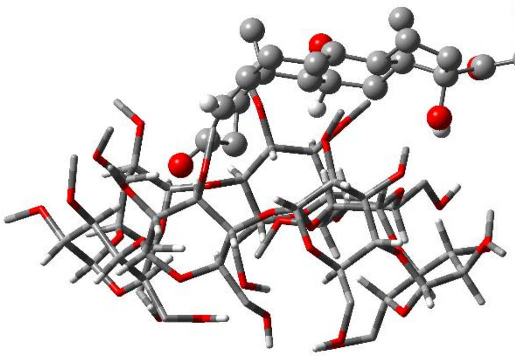

Hydrocortisone and **2,3-D-MeβCD** after 55.1 ns

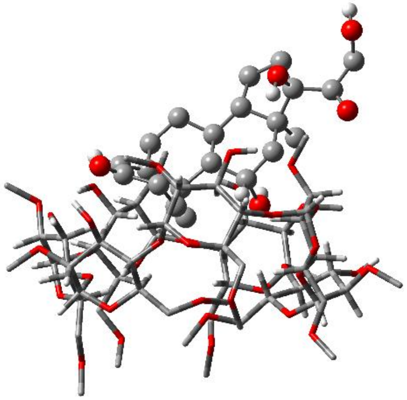

Hydrocortisone and **2,6-D-MeβCD** after 30.6 ns

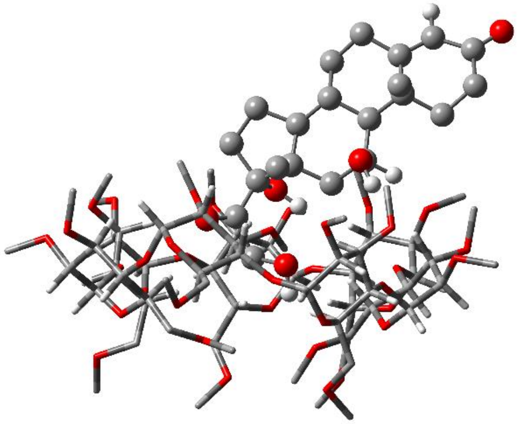

Hydrocortisone and **TMeβCD** after 164.7 ns

Figure S2: Snapshots of the moment and mechanism of Hydrocortisone entry into the CDs cavities.

Calculation methods for area and volume of cavity

The area of CDs cavity was calculated by the following equation:

$$A = \frac{\pi}{7}\sum_{i=1}^{7} r_i^2$$

Where r_i is the distance between each hydroxyl group and the center of O1 atoms, and the hydroxyl groups at 6- and 3-positions are used for representing the cavity area of primary and secondary hydroxyl rims, respectively.

The CD cavity has a shape that resembles a conical hourglass. As a result, we can approximate its volume by combining the volumes of the truncated cones located at the top and bottom of the cavity, as shown below.

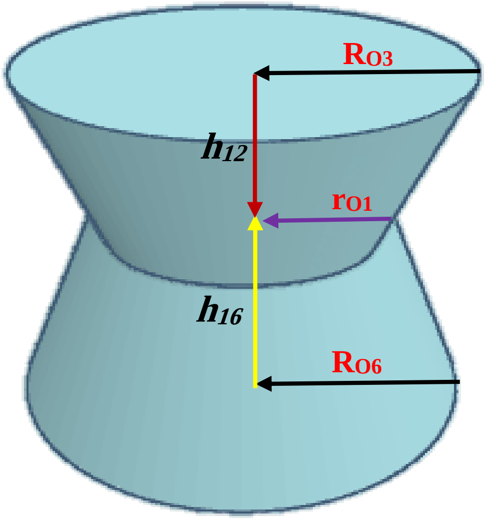

One can determine the volume of a truncated cone with a small radius "r", a large radius "R", and a height "h" by considering its geometry as follows:

$$Vc = \frac{1}{3}\pi h(r^2 + r \times R + R^2)$$

The radius of the O1 rim (Figure 1) was used as the small radius of the cones, while the radius of O3 rim and O6 rim was used as the large radius of the top and the bottom cone, respectively. The height, *h*, of the cones is sum of h12 and h16 listed in Table 1.

Table S2: Number of water molecules in different spheres inside the CDs cavity[a].

Distance / Systems	0-0.5 nm	0.5-1.0 nm	0-1.0 nm
**Water + βCD**	**9.9**	**90.1**	**100**
**Water + βCD + Hydrocortisone**	**0.17**	**83.46**	**83.63**
**Water + 2-MeβCD**	**5.74**	**85.57**	**91.31**
**Water + 2-MeβCD + Hydrocortisone**	**0.18**	**74.87**	**75.06**
**Water + 3-MeβCD**	**1.65**	**88.68**	**90.33**
**Water + 3-MeβCD + Hydrocortisone**	**0.14**	**75.69**	**75.83**
**Water + 6-MeβCD**	**3.12**	**88.88**	**92.00**
**Water + 6-MeβCD + Hydrocortisone**	**0.09**	**75.48**	**75.57**
**Water + 2,3-D-MeβCD**	**0.57**	**83.67**	**84.24**
**Water + 2,3-D-MeβCD + Hydrocortisone**	**0.27**	**68.30**	**68.57**
**Water + 2,6-D-MeβCD**	**1.36**	**83.45**	**84.81**
**Water + 2,6-D-MeβCD + Hydrocortisone**	**0.22**	**66.86**	**68.09**
**Water + TMeβCD**	**0.25**	**76.15**	**76.40**
**Water + TMeβCD + Hydrocortisone**	**0.07**	**60.50**	**60.57**

[a]All results were obtained from the last 10 % of the simulation time.

References:

1. W. Saenger, J. Jacob, K. Gessler, T. Steiner, D. Hoffmann, H. Sanbe, K. Koizumi, S.M. Smith, T. Takaha, Structures of the common cyclodextrins and their larger analogues beyond the doughnut, Chem. Rev. 98 (1998) 1787–1802.
2. C. Cézard, X. Trivelli, F. Aubry, F. Djedaïni-Pilard, F.-Y. Dupradeau, Molecular dynamics studies of native and substituted cyclodextrins in different media: 1. Charge derivation and force field performances, Phys. Chem. Chem. Phys. 13 (2011) 15103–15121.
3. S. Immel, Computer simulation of chemical and biological properties of saccharides: sucrose, fructose, cyclodextrins, and starch, (1995).
4. C. Betzel, W. Saenger, B.E. Hingerty, G.M. Brown, Topography of cyclodextrin inclusion complexes, part 20. Circular and flip-flop hydrogen bonding in. beta.-cyclodextrin undecahydrate: a neutron diffraction study, J. Am. Chem. Soc. 106 (1984) 7545–7557.
